# Risk Factors Associated with Reoperation for Bleeding following Liver Transplantation

**DOI:** 10.1155/2014/816246

**Published:** 2014-11-20

**Authors:** Maxwell A. Thompson, David T. Redden, Lindsey Glueckert, A. Blair Smith, Jack H. Crawford, Keith A. Jones, Devin E. Eckhoff, Stephen H. Gray, Jared A. White, Joseph Bloomer, Derek A. DuBay

**Affiliations:** ^1^University of Alabama at Birmingham, Birmingham, AL 35294, USA; ^2^Biostatistics Division, School of Public Health, University of Alabama at Birmingham, Birmingham, AL 35294, USA; ^3^Department of Anesthesia, University of Alabama at Birmingham, Birmingham, AL 35294, USA; ^4^Liver Transplant and Hepatobiliary Surgery, University of Alabama at Birmingham, 701 ZRB, 1530 3rd Avenue South, Birmingham, AL 35294-0007, USA; ^5^Transplant Hepatology, University of Alabama at Birmingham, Birmingham, AL 35294, USA

## Abstract

*Introduction*. This study's objective was to identify risk factors associated with reoperation for bleeding following liver transplantation (LTx). *Methods*. A retrospective study was performed at a single institution between 2001 and 2012. Operative reports were used to identify patients who underwent reoperation for bleeding within 2 weeks following LTx (operations for nonbleeding etiologies were excluded). *Results*. Reoperation for bleeding was observed in 101/928 (10.8%) of LTx patients. The following characteristics were associated with reoperation on multivariable analysis: recipient MELD score (OR 1.06/MELD unit, 95% CI 1.03, 1.09), number of platelets transfused (OR 0.73/platelet unit, 95% CI 0.58, 0.91), and aminocaproic acid utilization (OR 0.46, 95% CI 0.27, 0.80). LTx patients who underwent reoperation for bleeding had a longer ICU stay (5 days ± 7 versus 2 days ± 3, *P* < 0.001) and hospitalization (18 days ± 9 versus 10 days ± 18, *P* < 0.001). The risk of death increased in patients who underwent reoperation for bleeding (HR 1.89, 95% CI 1.26, 2.85). *Conclusion*. Reoperation for bleeding following LTx was associated with increased resource utilization and recipient mortality. A lower threshold for intraoperative platelet transfusion and antifibrinolytics, especially in patients with high lab-MELD score, may decrease the incidence of reoperation for bleeding following LTx.

## 1. Introduction 

Approaches to the perioperative management for liver transplantation have been adapted over time. Early experience with liver transplantation focused on the management of coagulopathy and involved extraordinary utilization of blood component therapy [1, 2]. Despite advances in anesthesia and surgical techniques manifesting in lower overall transfusion requirements [3, 4], bleeding is still the most frequent serious early complication following liver transplantation, occurring in approximately 20% of patients [5]. Coagulopathy management thus remains a major concern during orthotopic liver transplantation. Several recent and historic studies clearly demonstrate an association between intraoperative blood transfusion and mortality [6–9]. Interestingly, much variability exists between institutions with regard to coagulopathy management suggesting the need for more evidence to guide practice [2, 10, 11].

Postoperative bleeding can be life-threatening and requires reoperation in 10–15% of patients [5, 12] for hemorrhage control and/or hematoma evacuation. Reoperation for bleeding contributes to the overall mortality [13] and financial burden [14, 15] of liver transplantation. Many studies have attempted to identify risk factors associated with reoperation following liver transplantation; both donor and recipient variables have been reported [16, 17]. Previous research has identified that intraoperative estimated blood loss [18], increased intraoperative packed red blood cell (pRBC) transfusion [12, 18, 19], and total number of intraoperative blood products transfused are associated with reoperation following liver transplantation. In addition to massive transfusion, the UCLA group [20] found that recipient intraoperative vasopressor utilization, use of extended criteria donors, and recipient intraoperative glucose variability were also associated with reoperation for bleeding following liver transplantation. The problem with these sets of data is that they do little to help to predict, either before or during liver transplantation, the patients who are at increased risk for posttransplant bleeding. Identifying patients at increased risk of bleeding is important so a more aggressive approach to intraoperative coagulopathy management can be utilized. Furthermore, other than aggressive intraoperative glucose management, these studies did not identify modifiable risk factors associated with increased incidence of reoperation for bleeding.

The primary goal of this study was to identify risk factors associated with reoperation for bleeding following liver transplantation that may inform intraoperative coagulopathy management. Elucidation of such risk factors would aid in liver transplant coagulopathy planning by identifying patients in whom preemptive interventions are most appropriate.

## 2. Methods

### 2.1. Definition of Groups

Ethics approval for this study was obtained from the University of Alabama Institutional Review Board Protocol number X100310006. The requirement for written informed consent was waived by the Institutional Review Board.

A retrospective chart review was performed on all patients over 19 years of age who received a liver transplant at the University of Alabama at Birmingham between 2001 and 2012. Patients were identified from an internal transplant database that is used for clinical purposes. Patients were grouped based on whether or not a reoperation was performed for bleeding within 2 weeks of liver transplantation. The reoperation for bleeding group included any patient who required an unplanned exploratory laparotomy for either surgical management of active hemorrhage or evacuation of a hematoma. Operative notes were examined for the indication “hemoperitoneum, evacuation of hematoma, surgical management of active hemorrhage, or abdominal washout.” Other indications for reoperation such as bile leaks, vascular thrombosis, acute graft failure, or septicemia were excluded from the postoperative bleeding group.

### 2.2. Data Collected

Recipient demographics, comorbidities, selected laboratory values, operative details, and pertinent postoperative data were collected. Baseline demographics included age, gender, race, etiology of liver disease, lab-MELD scores, body mass index (BMI), and whether or not the patients were in the intensive care unit (ICU) immediately prior to transplant. Recipient comorbidities included the presence of diabetes, hypertension, dyslipidemia, chronic renal insufficiency, and coronary artery disease. Immediate pretransplant laboratory values included hematocrit, platelet count, and international normalized ratio (INR). Operative details included operative length, cold and warm ischemia time, blood products administered (red blood cells, fresh frozen plasma, platelets, and cryoglobulin), procoagulants administered (aminocaproic acid, Hospira, Lake Forest, IL, USA, and conjugated estrogens, Pfizer Canada, Kirkland, QC, Canada), epigastric surgical history (defined as a nonlaparoscopic, noncholecystectomy foregut intervention), vascular reconstruction (arterial conduit and/or mesenteric venous bypass), and retransplantation. Postoperative variables included intensive care unit length of stay, total posttransplant hospital length of stay, number of readmissions following transplant, graft failure, survival, and cause of death. Donor characteristics were summarized via the donor risk index (DRI) [21] which is a risk estimate of time to graft failure; variables included donor age, donor race, donor height, donor cause of death, type of donation (partial/split or whole), donor locations (regional, national, or local share), and donation after cardiac death.

### 2.3. Intraoperative Coagulopathy Management

The UAB liver transplant anesthesia utilizes both the thromboelastogram (TEG) functional assessment clot formation as well as INR, PTT, and platelet counts to manage intraoperative coagulopathy. The TEG assay is serially performed at the start of the transplant, during the anhepatic phase, and following reperfusion. If the* R* time is prolonged during the hepatectomy phase, conjugated estrogens are given at 1 mg/kg. Estrogens have been demonstrated to increase platelet adhesiveness [22, 23]. If the* R* time is within normal limits during the hepatectomy stage, then estrogens are given after reperfusion if the* R* time becomes prolonged then. Fresh frozen plasma (FFP) is used if estrogens fail to correct the* R* time. If FFP fails to adequately correct TEG parameters, hypofibrinogenemia exists, and surgical bleeding continues, cryoprecipitate is administered. Platelets (1 pheresed Unit) are administered if the platelet count is less than 50,000 and the surgical assessment is that of nonsurgical bleeding or if the maximum amplitude of the TEG is decreased. Epsilon aminocaproic acid (Amicar 2.5 gram iv) is used during the neohepatic stage (early reperfusion) if the TEG shows fibrinolysis.

### 2.4. Intensive Care Unit Posttransplant Coagulopathy Management

The UAB Surgical Intensive Care Unit utilizes a combination of the surgical drain effluent characteristics, INR, PTT, platelet counts, and thromboelastogram (TEG) functional assessment clot formation to manage posttransplant coagulopathy. In general, platelets and clotting factors are only administered in the setting of active bleeding, sanguineous drain output, a decreasing hematocrit, or extreme laboratory values.

### 2.5. Statistical Analysis

Descriptive statistics (sample means and variances) for continuous variables (age, body mass index, MELD score, and donor risk index) were calculated to provide measures of central location and variability around the mean. For categorical variables (race, comorbidities, etc.), sample proportions falling within each category were calculated. To test the primary hypothesis that long-term survival varied by reoperation for bleeding, Log-Rank tests were used, and Kaplan-Maier curves were constructed to examine/compare liver transplant survival distributions. Multivariable Cox regression analyses were performed to control for potentially confounding variables. Demographics and comorbidities were compared between groups (reoperation for bleeding versus control group) using Chi-Square tests for categorical variables and two-sample *t*-tests for continuous variables. To examine which demographics and factors were associated with odds of reoperation for bleeding, multivariable logistic regression models were developed. All analyses were conducted using SAS 9.3 (Cary NC) and statistical significance was defined as a *P* value less than or equal to 0.05.

## 3. Results

### 3.1. Baseline Demographics (Table 1)

The reoperation for bleeding group consisted of 101/928 (10.8%) of liver transplant patients. The mean age, race, BMI, and etiology of liver disease were not statistically significant between the reoperation for bleeding group and control group. There was a trend towards fewer males in the reoperation for bleeding group, but this did not reach statistical significance. The average lab-MELD score at the time of transplantation was significantly higher in the reoperation for bleeding group compared to the control group (23.8 ± 8.1 versus 20.4 ± 7.9, *P* < 0.001). Similarly, a significantly higher percentage of the patients from the reoperation for bleeding group had been in the ICU at the time of transplantation compared to the control group (23.2% versus 12.9%, *P* = 0.005). The immediate pretransplant hematocrit (26.0% ± 8.0 versus 25.8% ± 8.9, *P* = 0.80) and platelet counts (88.8 versus 79.9, *P* = 0.14) were similar between groups whereas the INR was elevated in the patients from the reoperation for bleeding group compared to the control group (2.1 ± 0.9 versus 1.9 ± 0.9, *P* = 0.04).

The reoperation for bleeding was performed within 1 week in 79/101 (78%) of the patients and after 1 week in the remaining 22/101 (22%). An active bleeding source was identified in 27/101 (27%) patients whereas in the remaining 74/101 (73%) patients the bleeding etiology was diagnosed as coagulopathic bleeding. Identifying an active bleeding source was more common when the reoperation for bleeding was performed within 48 hours after transplant (<48 hr 41% versus >48 hr 12%, *P* = 0.002).

### 3.2. Donor and Operative Characteristics

There were no differences in donor age, donor race, donor cause of death, or donor height between groups. The only difference between groups was a significantly higher percentage of organ grafts from regional donor in the reoperation for bleeding versus control group (31.0% versus 18.8%, *P* = 0.0025). Despite the difference in utilization of regional donors, there was no significant difference in the donor risk index (DRI) between reoperations for bleeding versus control group (1.6 ± 0.4 versus 1.5 ± 0.4, *P* = 0.61).

There were no differences in operation length, cold ischemia time, or warm ischemia time between groups (Table 2). The number of units of platelets transfused intraoperatively was significantly lower in the reoperation for bleeding group compared to the control group (1.1 ± 1.4 versus 1.9 ± 3.7, *P* < 0.001). There were no statistical differences in units of red blood cells transfused between groups when analyzing as a continuous variable (4.3 ± 4.2 versus 3.7 ± 3.5, *P* = 0.19). Because mass red blood cell transfusion has been indicated as a risk factor for reoperation in previous studies [12, 16–20], we further analyzed red blood cells transfusion by categorizing units of red blood cells transfused into quartiles and comparing these groups but again demonstrated no association with reoperation (*P* = 0.31). Similarly, there were no differences in units of fresh frozen plasma transfused between groups when analyzing as a continuous variable (1.8 ± 2.6 versus 1.5 ± 1.8, *P* = 0.19) or as a quartile categorical variable (*P* = 0.28). Cryoprecipitate was used infrequently but statistically more often in the reoperation for bleeding group compared to the control group (4.5% versus 1.3%, *P* = 0.05). There was a trend toward less estrogen usage in the reoperation for bleeding group, although this did not reach statistical significance (27.0% versus 35.4%, *P* = 0.11). Aminocaproic acid was administered less often in the reoperation for bleeding group compared to the control group (20.2% versus 30.2%, *P* = 0.05). The frequency of epigastric surgical history, venous or arterial reconstruction, and retransplant rates was similar in both groups (Table 2).

The platelet count immediately following liver transplantation was not statistically different between groups (77.98 ± 33.21 versus 80.74 ± 41.33, *P* = 0.63). The platelet count was significantly lower in the reoperation for bleeding group by 24 hours (72.69 ± 30.52 versus 83.76 ± 45.59, *P* = 0.01) and by 96 hours (52.85 ± 24.30 versus 65.83 ± 44.65, *P* = 0.001) following liver transplantation. There were no differences in INR immediately postoperatively or at 24 or 48 hours. The median time to INR <2 was 2 days in both groups.

### 3.3. Analysis of Factors Associated with Reoperation for Bleeding (Table 3)

**Figure 1:**
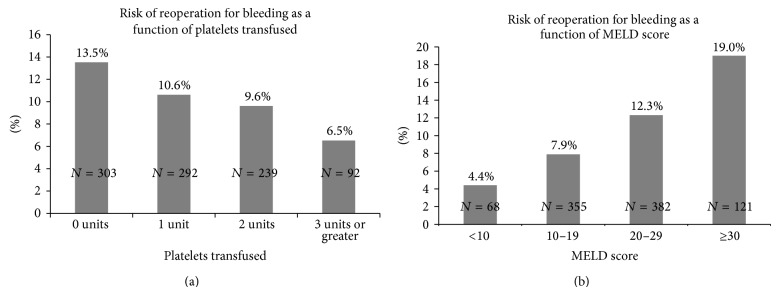
(a) Risk of reoperation as a function of platelets transfused. The risk of reoperation was highest in the group of liver transplant recipients that did not receive platelet transfusion during the transplant operation (13.7% risk estimate). Conversely, the lowest risk of reoperation for bleeding was observed in the group of patients that received the highest number of platelets transfused (5.9% risk estimate). (b) Risk of reoperation for bleeding as a function of lab-MELD score. There was a stepwise increase in the risk of reoperation for bleeding as recipient lab-MELD score increased. The lowest risk estimate was for a lab-MELD ≤10 (5.4% estimate) whereas the highest risk estimate was for a lab-MELD >30 (19.4% estimate).

A univariate and multivariable analysis of factors associated with reoperation for bleeding following liver transplant was performed. On univariate analysis, the following variables were associated with reoperation for bleeding following liver transplant: lab-MELD score (OR 1.05/point, 95% CI 1.03, 1.08, *P* < 0.0001), ICU immediately prior to transplant (OR 2.05, 95% CI 1.23, 3.15, *P* = 0.0057), platelets transfused during transplantation (OR 0.87/unit, 95% CI 0.76, 0.99, *P* = 0.0373), and utilization of aminocaproic acid (OR 0.59, 95% CI 0.34, 0.99, *P* = 0.05). A multivariable model was fitted to determine the association with reoperation for bleeding (Table 3). All variables statistically significant on the univariate analysis as well as the following clinically important variables were entered into the model: surgeon, recipient history of epigastric surgery, donor risk index, warm ischemia time, duration of the operation, units pRBC transfused, and units FFP transfused. Recipient lab-MELD score (OR 1.06/MELD unit, 95% CI 1.03–1.09, *P* < 0.0001), number of platelets transfused (OR 0.73/platelet unit, 95% CI 0.58–0.91, *P* = 0.004), and utilization of aminocaproic acid (OR 0.46, 95% CI 0.27, 0.80, *P* = 0.006) were statistically associated with reoperation for bleeding following liver transplant.  Figure 1(a) illustrates the risk of reoperation as a function of platelets transfused including the mean number of platelets before transplant, immediately after transplant, and 24 hours after transplant.  Figure 1(b) illustrates the risk of reoperation for bleeding as a function of lab-MELD score quartiles.

### 3.4. Outcome Characteristics (Figure 2)

The risk of death was increased in liver transplant recipients who underwent reoperation for bleeding versus those that did not (HR 1.89, 95% CI 1.26–2.85). The survival curves continue to separate until about 9 months postoperatively, at which time the difference in survival becomes relatively constant. Death prior to discharge was observed in 4.95% of the patients reoperated for bleeding versus 2.54% of the control group (*P* = 0.17). There was significantly higher mortality rates within 12 months of transplantation observed in the reoperation for bleeding group (15.84% versus 7.78%, *P* = 0.007). The causes of death in each group are presented in Table 4.

There were no differences in 1-, 3-, or 5-year survival based upon platelet transfusion (no platelets transfused, *n* = 301: 93%, 87%, 81% versus 1 or more units of platelets transfused, *n* = 627: 90%, 84%, 78%, *P* = 0.26). Liver transplant patients who underwent reoperation for bleeding had a longer total ICU stay (5 days ± 7 versus 2 days ± 3, *P* < 0.001) and hospitalization (18 days ± 9 versus 10 days ± 18, *P* < 0.001) (data expressed as median ± interquartile range). There were no statistical differences in the number of readmissions between the reoperations for bleeding and control group.

## 4. Discussion

There is need for more clinical studies to guide intraoperative management of coagulopathy during liver transplantation [2, 10, 11]. Approaches vary from preventive practices that involve decision-making based on laboratory values and some type of “recipe” for product administration associated with each unit of blood transfused, to the other extreme of not even addressing the coagulopathy until the new liver is reperfused. Even when there is a clear-cut coagulopathy present, it is often not obvious what to administer to ameliorate nonsurgical bleeding. Is it the best approach to treat an INR and platelet count or base decisions on a functional TEG assay? What threshold values should be treated? The holy grail of liver transplant coagulopathy is what to administer, when, and how much? A brief discussion with a group of transplant anesthesiologists/surgeons will quickly reveal the disparity in coagulopathy approaches with a lot of “based upon my training” or “in my experience” anecdotes, variability well described in the literature [10].

The goal of treating coagulopathy is preventing bleeding, especially postoperative bleeding requiring a second laparotomy. Reoperation is a (negative) quality measure tracked and reported by UNOS that is associated with significantly increased resource utilization [14, 15]. Previous studies examining risk factors for reoperation for bleeding following liver transplant essentially report that difficult liver transplant operations associated with high blood product transfusion rates are associated with increased rates of reoperation [12, 13, 20]. This study, to the best of our knowledge, is the first to demonstrate a stepwise association between rising lab-MELD score and likelihood of reoperation for bleeding (Figure 1(b)). The odds of a reoperation for bleeding increased 1.06 per MELD point. The univariate analysis also suggested an association between ICU status and risk of reoperation for bleeding as well as regional donors and reoperation for bleeding. These associations, however, were not significant on multivariable analysis, probably because these variables are correlated with recipient high lab-MELD scores. The association of high lab-MELD score with reoperation for bleeding may be used to help to risk-stratify coagulopathy approaches prior to the transplant operation. For example, a more aggressive approach to perioperative coagulopathy management may be indicated including the prophylactic use of antifibrinolytics [24] and lower thresholds to administer blood products and concentrated factors [25].

Perhaps the most interesting finding of this study is that intraoperative platelet administration was associated with a significantly lower likelihood of reoperation for bleeding following liver transplantation. For each unit of platelets transfused, the odds of reoperation for bleeding decreased by 0.73.  Figure 1(a) demonstrates that the highest risk of reoperation for bleeding was observed in the group that received no platelet transfusion, a liver transplant group that may have been predicted to be uncomplicated operations as evidenced by the lack of perceived need for platelet transfusion. However, this group was associated with the highest rates of reoperation for bleeding. Conversely, those patients that received more than 1 unit of platelets may have been subjected to operations in which the surgeon was struggling to “dry up the field.” Somewhat, surprisingly, there was no statistical association between plasma administration and decreased reoperation for bleeding, similar to that reported by Massicotte and the Montreal Liver Transplant group [26]. This observation is also similar to findings by Giannini et al. who reported that invasive procedure-related bleeding in cirrhotics listed for liver transplant was only observed in patients with severe thrombocytopenia, whereas significant coagulopathy was not associated [27].

Nonutilization of aminocaproic acid also was an independent risk factor predicting reoperation for bleeding. The decision to not administer antifibrinolytics can best be described as a practice variable amongst surgeons and anesthesiologists which is supported by reports associating thromboembolic events with antifibrinolytic administration [28]. However, the use of antifibrinolytics is generally considered safe and supported by multiple studies showing prophylactic or therapeutic administration decreases bleeding and reduces blood product administration [29–33]. Here we expand on these studies by demonstrating an association between decreased reoperation for bleeding and aminocaproic acid administration as a new finding.

Administration of blood products is not a benign intervention, however. There are reports that platelet transfusions are associated with decreased survival following liver transplantation [34, 35]. The Netherlands liver transplant group reported a higher hazard of death in transplant recipients that received platelets (HR 1.37/unit of platelets) compared to those patients that did not receive any platelets [35]. Our data did not demonstrate a similar relationship between platelet administration and mortality. In contrast, this study demonstrates that reoperation is the main factor associated with increased mortality after liver transplant. The increased hazard of death was 1.89 in liver transplant recipients who underwent reoperation for bleeding. These sets of data suggest that interventions that may decrease reoperations for bleeding may decrease posttransplant mortality, even if these interventions involve administering blood products. Another interesting finding is that the risk of death in patients that underwent reoperation for bleeding persisted for 1 year after transplant. Deaths due to infection and recurrent hepatitis C were numerically higher in the reoperation for bleeding group compared to the control group.

This study is limited by the fact that it is a single center study consisting of the results of a dedicated 4-member liver transplant anesthesia team and only 4 transplant surgeons. The results are influenced by the internal practice patterns by the liver transplant anesthesia and surgical team, which may limit generalizability. For example, the beneficial effect of platelets or aminocaproic acid may not be as important in programs with a more liberal approach to managing intraoperative coagulopathy. Finally, this study does not identify threshold values for platelet administration and the data do not suggest optimal timing of platelet administration.

In conclusion, this study demonstrates that reoperation for bleeding following liver transplantation is associated with significant resource utilization and increased postoperative mortality. The only modifiable risk factors associated with decreased risk of reoperation for bleeding were intraoperative platelet administration and aminocaproic acid utilization. Our data suggests that adequate platelet administration and use of antifibrinolytics may be key interventions in the management of liver transplant associated coagulopathy, especially in recipients with a high lab-MELD score. Utilization of this knowledge may be helpful for liver transplant anesthesia protocols designed to minimizing the incidence and impact of reoperation for bleeding following liver transplantation.

## Figures and Tables

**Figure 2:**
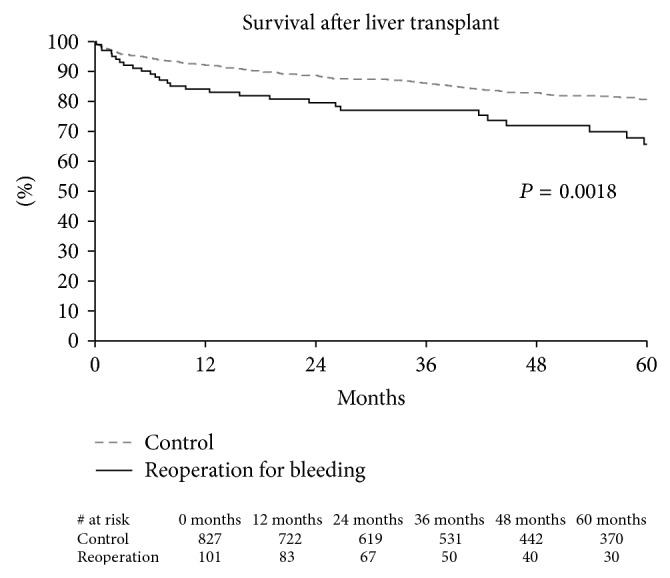
Survival after liver transplant stratified by reoperation for bleeding. Decreased survival was observed in the group of liver transplant recipients that underwent reoperation for bleeding (*P* = 0.0018).

**Table 1:** Baseline demographics.

Variable	Reoperation for bleeding	Control group	*P* value
Group	*n* = 101	*n* = 827	
Age (years)	51.7 ± 11.9	53.5 ± 9.7	0.16
Male (%)	54.5	63.4	0.08
Race			
Caucasian %	81.2	85.9	0.43
African-American %	14.9	10.8	
BMI	29.6 ± 6.3	29.0 ± 6.4	0.43
Etiology of liver disease (%)			
Hepatitis C	44.6	36.4	0.11
NASH/cryptogenic	15.8	20.6	0.26
Laennec's	9.0	12.7	0.28
Cholestatic (PBC, PSC)	9.9	11.9	0.55
Other^a^	8.9	9.0	0.98
MELD Score	23.8 ± 8.1	20.4 ± 7.9	<0.001
ICU prior to liver transplant (%)	23.2	12.9	0.005
Comorbidities			
Dyslipidemia	12.9	13.6	0.85
Diabetes	21.8	26.3	0.33
CAD	5.0	3.5	0.47
CRI	10.9	7.5	0.24
Hypertension	51.5	47.3	0.42
Baseline lab values			
Platelet count (×10^9^/L)	88.8	79.9	0.14
Hematocrit	26.0 ± 8.0	25.8 ± 8.9	0.80
INR	2.1 ± 0.9	1.9 ± 0.9	0.04
Split liver transplant (%)	1.0	0.25	0.30

Data presented as percent or mean ± standard deviation.

^a^Other diagnoses include autoimmune hepatitis, alpha 1 antitrypsin deficiency, hemochromatosis, fulminant liver failure, Budd-Chiari syndrome, Wilson's disease, polycystic liver disease, nonhepatocellular carcinoma neoplastic disease, sarcoidosis, secondary biliary cirrhosis, Caroli's disease, and cystinosis.

NASH: nonalcoholic steatohepatitis; PBC: primary biliary cirrhosis; PSC: primary sclerosing cholangitis; MELD: Model for End-Stage Liver Disease; BMI: body mass index; CAD: coronary artery disease; CRI: chronic renal insufficiency; INR: international normalized ratio.

**Table 2:** Characteristics of the liver transplant operation.

Variable	Reoperation for bleeding	Control group	*P* value
Operation length (min)	269.8 ± 119.6	278.2 ± 104.9	0.50
Cold ischemia time (min)	405.5 ± 166.9	390.8 ± 174.4	0.41
Warm ischemia time (min)	49.3 ± 45.7	47.9 ± 18.4	0.46
Transfusion^a^			
Red blood cells (units)	4.3 ± 4.2	3.7 ± 3.5	0.19
Fresh frozen plasma (units)	1.8 ± 2.6	1.5 ± 1.8	0.19
Platelets (units)	1.1 ± 1.4	1.9 ± 3.7	<0.001
Cryo administered (%)	4.49	1.33	0.05
Last fibrinogen level	151.8 ± 107.4	124.6 ± 49.6	0.22
Estrogen administered (%)	26.97	35.37	0.11
Amicar administered (%)	20.22	30.19	0.05
Epigastric surgical history (%)	24.8	24.2	0.91
Vascular reconstruction^b^ (%)	1.0	4.4	0.17
Retransplant (%)	5.0	4.3	0.80

Data presented as percent or mean ± standard deviation.

^a^Blood products transfused only during the liver transplant operation do not include data on reoperation for bleeding.

^b^Includes both arterial conduits and mesenteric venous bypass grafts.

**Table 3:** Univariate and multivariable analysis of factors associated with reoperation for bleeding following liver transplantation.

Variable	Univariate analysis			Multivariable analysis		
	Odds ratio	95% CI	*P* value	Odds ratio	95% CI	*P* value
Lab-MELD	1.05	1.03, 1.08	<0.0001	1.06	1.03, 1.09	<0.0001
Surgical history	0.93	0.42, 2.10	0.87			
ICU Pre-LTx	2.05	1.23, 3.15	0.006			
WIT (Min)	1.00	0.99, 1.01	0.48			
OR length	0.99	0.98, 1.00	0.13			
DRI	0.85	0.48, 1.52	0.58			
pRBC	1.04	0.99, 1.10	0.13			
FFP	1.09	0.99, 1.20	0.08			
PLT	0.87	0.76, 0.99	0.04	0.73	0.58, 0.91	0.004
Estrogen	0.67	0.41, 1.10	0.12			
Aminocaproic acid	0.59	0.34, 0.99	0.05	0.46	0.27, 0.80	0.006

MELD: Model for End-Stage Liver Disease; ICU: intensive care unit; LTx: liver transplantation; WIT: warm ischemia time; OR: operating room; DRI: donor risk index; pRBC: packed red blood cells; FFP: fresh frozen plasma; PLT: platelet; LTx: liver transplantation.

**Table 4:** Cause of death.

Variable	Reoperation for bleeding	Control group
Number of deaths	28 (27.7%)	137 (16.5%)
Cause of death^a^		
Infection	35.7%	25.6%
Cancer	3.6%	19.0%
Cardiac disease	3.6%	10.2%
Recurrent HCV	21.4%	9.5%
Other^b^	35.7%	35.8%
Death prior to discharge	4.95%	2.54%
Death within 12 months of LTx	15.84%	7.78%

^a^Proportions for cause of death are calculated on the subset of 165 individuals (137 with no hemorrhage, 28 with hemorrhage) that died within 60 months of transplant. All other proportions are based on total sample size.

^b^Other causes of death include chronic rejection, cerebrovascular accident, gastrointestinal bleeding, chronic renal failure, graft versus host disease, suicide, and recurrent Budd-Chiari syndrome.

HCV: hepatitis c virus; LTx: liver transplantation.
